# AXL targeting by a specific small molecule or monoclonal antibody inhibits renal cell carcinoma progression in an orthotopic mice model

**DOI:** 10.14814/phy2.15140

**Published:** 2021-12-08

**Authors:** Tony J. Chen, Piotr Mydel, Małgorzata Benedyk‐Machaczka, Marta Kamińska, Urszula Kalucka, Magnus Blø, Jessica Furriol, Gro Gausdal, James Lorens, Tarig Osman, Hans‐Peter Marti

**Affiliations:** ^1^ Department of Clinical Medicine University of Bergen Bergen Norway; ^2^ Department of Clinical Science University of Bergen Bergen Norway; ^3^ Department of Microbiology Jagiellonian University Krakow Poland; ^4^ BerGenBio ASA Bergen Norway; ^5^ Department of Biomedicine Centre for Cancer Biomarkers Norwegian Centre of Excellence University of Bergen Bergen Norway; ^6^ Department of Medicine Haukeland University Hospital Bergen Norway

**Keywords:** bemcentinib, orthotopic RCC, tilvestamab

## Abstract

AXL tyrosine kinase activation enhances cancer cell survival, migration, invasiveness, and promotes drug resistance. AXL overexpression is typically detected in a high percentage of renal cell carcinomas (RCCs) and is strongly associated with poor prognosis. Therefore, AXL inhibition represents an attractive treatment option in these cancers. In this preclinical study, we investigated the antitumor role of a highly selective small molecule AXL inhibitor bemcentinib (BGB324, BerGenBio), and a newly developed humanized anti‐AXL monoclonal function blocking antibody tilvestamab, (BGB149, BerGenBio), in vitro and an orthotopic RCC mice model. The 786‐0‐Luc human RCC cells showed high AXL expression. Both bemcentinib and tilvestamab significantly inhibited AXL activation induced by Gas6 stimulation in vitro. Furthermore, tilvestamab inhibited the downstream AKT phosphorylation in these cells. The 786‐0‐Luc human RCC cells generated tumors with high Ki67 and vimentin expression upon orthotopic implantation in athymic BALB/c nude mice. Most importantly, both bemcentinib and tilvestamab inhibited the progression of tumors induced by the orthotopically implanted 786‐0 RCC cells. Remarkably, their in vivo antitumor effectiveness was not significantly enhanced by concomitant administration of a multi‐target tyrosine kinase inhibitor. Bemcentinib and tilvestamab qualify as compounds of potentially high clinical interest in AXL overexpressing RCC.

## Graphical abstract

AXL tyrosine kinase activation enhances cancer survival and invasiveness, and its overexpression is strongly associated with poor prognosis in advanced renal cell carcinoma. In our preclinical study, we investigated the anti‐tumor effect of two AXL inhibitors, bemcentinib and tilvestamab, in vitro and in orthotopically implanted 786‐0 RCC cells in mice. The results showed that treatment with the AXL inhibitors successfully inhibit AXL activation in vitro and significantly reduce tumor growth in orthotopically implanted RCC, thus support their clinical relevance and warrant future clinical testing.

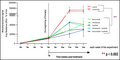

**New & Noteworthy**

Upregulation of AXL receptors is associated with a spectrum of characteristics frequently observed in renal malignancies. We found that AXL‐targeted agents bemcentinib and tilvestamab effectively inhibit AXL activation in vitro and RCC cells growth in an orthotopic implanted mice model. This supports their clinical relevance and warrants future clinical testing.

## 1 INTRODUCTION

Renal Cell Carcinoma (RCC) is a urological cancer accounting for approximately 3%–5% of all malignancies worldwide. Its incidence rate has steadily increased in the last decades, mostly due to the growing prevalence of risk factors such as smoking, hypertension, and obesity (Ljungberg et al., 2019; Yuan et al., 1998).

The most common histological type of RCC is clear cell renal cell carcinoma (ccRCC), and treatment is based on partial or total nephrectomy in localized/localized advanced RCC, and systemic therapy in metastatic RCC (mRCC) (Ljungberg et al., 2019). Nevertheless, prognosis often remains poor. Around 20%–30% of patients have mRCC at initial diagnosis (Ljungberg et al., 2019). According to the International mRCC database consortium (IMDC), the median overall survival rate in mRCC is of 27 months in IMDC‐categorized intermediate‐risk group and 8.8 months in the high‐risk group (Heng et al., 2009). Moreover, a 5‐year relapse rate of 30%–40% has been observed in patients who underwent surgical nephrectomy for localized advanced RCC (Janowitz et al., 2013). Approximately 30% of mRCC patients do not respond to the standard treatment with tyrosine kinase inhibitors due to intrinsic resistance, resulting in unfortunate clinical outcome (Porta et al., 2012). Therefore, new therapeutic strategies are urgently required.

AXL receptor, a transmembrane kinase receptor and member of TYRO3, AXL and MERTK (TAM) family, was first characterized in chronic myeloid leukemia in 1991 and thereafter has been identified in a variety of malignancies such as breast, esophageal, and non‐small cell lung cancers (NSCLC), as well as in RCC (Chung et al., 2003; Gay et al., 2017). Since its discovery, AXL has been shown to be involved in a wide range of signaling pathways such as PI3/AKT, MAPK, and SNAIL/EMT, promoting tumor cell survival and proliferation, as well as tumor migration and invasiveness (Byers et al., 2013; Han et al., 2013; Sainaghi et al., 2005; Zhang et al., 2013). In addition, AXL activation may promote immune suppression through SOC1/3 signaling, enabling tumor evasion (Gay et al., 2017).

AXL upregulation is associated with aggressive and drug‐resistant RCC and is regarded as a poor prognosis marker (Gay et al., 2017; Yu et al., 2015; Zucca et al., 2018), thus identifying this protein as a potential target of anticancer therapy. Most recently, AXL expression in advanced RCC has also been associated with resistance to immunological checkpoint blockade (Terry et al., 2021).

Bemcentinib (R428/BGB324) is a selective small molecule AXL kinase inhibitor. Treatment with this drug has shown to induce cancer cell apoptosis, inhibit invasiveness, and alleviate drug resistance in breast cancer, NSCSC, and esophageal cancer xenografted models (Holland et al., 2010; Wnuk‐Lipinska et al., 2014; Yang et al., 2019). Based on these findings, bemcentinib is currently being tested in phase 2 clinical trials for various types of cancers such as melanoma, NSCSC, pancreatic, and AML (Zhu et al., 2019). However, bemcentinib's in vivo role in experimental renal cell carcinoma has only been explored in subcutaneous heterotopic RCC models (Yu et al., 2015).

In this study, we evaluated the effects of two different AXL inhibitors, a small molecule bemcentinib (BGB324, BerGenBio) and a newly developed humanized anti‐AXL antibody tilvestamab (BGB149, BerGenBio) (Blø et al., 2020) alone or in combination with multi‐targeted tyrosine kinase inhibitor (Sunitinib, Pfizer) in an orthotopic RCC model.

## 2 MATERIALS AND METHODS

### 2.1 Renal cell cancer cells

Human cell lines 786‐0 (RRID: CVCL_1051) and 786‐0‐Luc (RRID: CVCL_J240) were purchased from the Japanese Collection of Research Bioresources (JCRB) Cell Bank. These cells were maintained in RPMI‐1640 medium supplemented with 10% fetal bovine serum and with 100 units/ml penicillin and 100 µg/ml streptomycin at 37°C with 5% CO_2_. Cells were subcultured after reaching confluence using 0.25% trypsin. Washings were performed using PBS.

### 2.2 Gene expression studies

Real‐time reverse transcription quantitative polymerase chain reaction (RT‐qPCR) was used to verify AXL gene expression in 786‐0‐Luc cells. Briefly, total cellular RNA was extracted from lysed cells using Trizol reagent (Thermofisher, 15596026) following the manufacturer's protocol. cDNA was obtained using Applied Biosystems^TM^ reverse transcription kit (4368814). PCR was performed using 5ʹGTGGGCAACCCAGGGAATATC 3ʹ forward and 3ʹGTACTGTCCCGTGTCGGAAAG 5ʹ reverse AXL‐specific primers (Sigma) and a pre‐formulated master mix (Applied Biosystems^TM^, A25779). GAPDH gene expression was used as a control.

The RT‐qPCR was performed on a CFX96 Touch Real‐Time PCR (Bio‐Rad) under the following conditions: hold at 95°C for 15 min, and 40 amplification cycles, each including melt at 95°C for 30 s, followed by annealing/extension at 58°C for 30 s with a final step incubation at 72°C for 30 s. Expected size of the product of interest was 234 bp, and PCR products were visualized on 2% agarose gels. Samples were analyzed in triplicates.

### 2.3 Western blot analysis of AXL

The AXL protein was detected by western blot analysis. Briefly, 786‐0 and 786‐Luc cells were lysed with RIPA buffer (Roche), and proteins were separated by SDS‐PAGE and transferred onto PVDF membranes.

Membranes were blocked with 5% skim milk in TTBS buffer (0,05% Tween‐20, 50 mM Tris, 150 mM NaCl) for 2 h at room temperature (RT), and thereafter incubated with goat anti‐AXL antibodies 1:200 (AF154, R&D Systems) at 4°C overnight. Subsequently, membranes were incubated with HRP‐conjugated anti‐goat IgG (A5420, Sigma, 1:3000) for 2 h at RT. Blots were then developed using Pierce ECL western blotting substrate and signals were detected on photographic films (Kodak). Technical replicates were performed by duplicate in each sample.

### 2.4 Exposure of 786‐O cells to AXL inhibitors in vitro

786‐O cells were placed in starvation medium (0.35% BSA) for 4 h and were then treated with tilvestamab (BGB149, BerGenBio, Norway), a humanized AXL monoclonal antibody (4.7 μg/ml), or bemcentinib (BGB324, BerGenBio, Norway) (250 µM) for 1 h prior to stimulation with Gas6‐Fc (Evitria, Schlieren, Switzerland) for 20 min. IgG and dimethyl sulfoxide (DMSO) were used as a control for tilvestamab and bemcentinib, respectively. After treatment, cells were lysed with RIPA buffer, and lysates were analyzed by a pAXL‐Y866 ELISA (BerGenBio) described below.

### 2.5 Phospho‐AXL‐Y866 ELISA

Capture antibody (mouse anti‐AXL, BerGenBio#5F11) was diluted to 3.6 μg/ml in coating buffer (0.2 M sodium carbonate, pH 9.4). In total, 100 μl/well was added to a 96‐well microplate, the plate was sealed and incubated overnight at RT. Wells were washed three times with wash buffer (TBS, 0.5% Tween) using an automated plate washer. Wells were blocked by adding 300 μl of blocking reagent (5% dry milk in TBS) to each well and incubated for 3 h at 37°C with plate sealer. Wells were washed as described. Hundred microliters of cell sample prepared in RIPA buffer, with phosphatase and protease inhibitors, was added to wells in duplicates. Plates were sealed and incubated overnight at 4°C. Wells were washed twice before adding 100 μl/well of detection antibody (rabbit polyclonal pAXL‐Y866, BerGenBio, 100 ng/ml, diluted in blocking reagent). Plates were sealed and incubated for 2 h at RT. Wells were washed as above, and 100 μl/well of secondary detection antibody (goat‐anti rabbit HRP, Jackson Labs, 111‐0350144, diluted 1:16,000 in blocking reagent) was added. Plates were sealed and incubated for 2 h at RT. Wells were washed as above, and 100 μl/well of substrate solution (equal volumes SuperSignal ELISA Femto substrate solution A and B, Thermo Fisher Scientific) was added and the plate was placed on a shaker for 1 min. Recording of luminescence was performed on a microplate reader, collecting all wavelengths for total light output. Results from tilvestamab (*n* = 6) and bemcentinib (*n* = 4) experiments were expressed as ratio over IgG control and DMSO, respectively.

### 2.6 Phospho‐AKT‐S473 ELISA in tilvestamab‐treated cells

Three biological replicates were performed per group to measure phospho‐AKT‐S473. AKT Multispecies InstantOne™ ELISA Kit (Thermo Fisher Scientific, 85‐86046‐11) was used. The manufacturer´s instructions were followed. Phospho‐AKT absorbance was measured at 450 nm with background correction at 540 nm.

### 2.7 Tumor implantation in mice

Female, 8‐week‐old BALB/c athymic nude mice (Janvier Labs, Le Genest‐Saint‐Isle, France) were used to evaluate the antitumor activity of novel AXL inhibitors in vivo. Animals were housed and maintained in individually ventilated cages under a 50%–60% humidity, 12‐h light/dark cycle, at 22 ± 2°C, in SPF conditions at the Faculty of Biochemistry, Biophysics and Biotechnology of the Jagiellonian University in Krakow, Poland. All experimental procedures were reviewed and approved by the II Regional Ethics Committee on Animal Experimentation, Krakow, Poland (approval no: 220/2018).

Three percent to four percent of isoflurane (Baxter, Deerfield, IL, USA) was used to induce anesthesia in mice and maintained at 1%–2% with 0.5 l/min air flow. The planned incision area was first disinfected and then injected subcutaneously (*sc*) with a local analgesic (buprenorphine 0.1 mg/kg body weight). Thereafter, a 0.3 cm surgical incision was made and the kidney was exposed through the incision. 786‐0‐Luc cell suspensions (2.5 × 10^6^/20 µl, solution composed of 1:3 PBS and Matrigel Matrix [Corning, Ref 356237]) were implanted under kidney capsule using 0.5 ml syringe with 29‐gauge needle. After implantation, the peritoneum, muscles, and skin were stitched, and mice placed in a heating incubator (30–33°C). Buprenorphine 0.1 mg/kg was given sc 6 h post‐operation and then every 8 h for 24 h.

### 2.8 Drug treatment in orthotopic RCC

#### 2.8.1 In vivo study I

After 4 weeks of initial tumor growth (~50 mm³), mice with similar tumor volumes were randomized into seven to eight animals per group prior to treatment. Bemcentinib was given through oral gavage at the dose of 50 mg/kg/mouse every 12 h. The solvent of bemcentinib, methylcellulose 2%, was given at same volume to the control group.

Tumor growth was measured after 10, 17, 24, and 34 days of therapy using Lumina in vivo imaging system (IVIS; PerkinElmer, Waltham, MA, USA). Briefly, 15 mg/kg bodyweight D‐luciferin (XenoLight D‐luciferin potassium salt; PerkinElmer, Waltham, MA, USA) was injected into the peritoneal cavity and bioluminescence measurement was performed on anesthetized animals after 10 min. Tumor growth was also evaluated at each time point by ultrasound using 3D‐USG VEVO 2100, MS550D transducer (FUJIFILM VisualSonics, Inc., Toronto, ON, Canada). Results were analyzed in 3D mode using VEVO 2100 Software (FUJIFILM VisualSonics, Inc., Toronto, ON, Canada).

#### 2.8.2 In vivo study II

A second series of orthotopic experiments with inclusion of tilvestamab and sunitinib (Pfizer Inc. NY USA) was subsequently conducted. After 8 weeks of tumor growth, animals were randomized into seven groups (six to seven animals per group). Three groups were treated with single‐agent: bemcentinib––orally 50 mg/kg/mouse every 12 h, tilvestamab––with intraperitoneal (IP) injection twice a week at 30 mg/kg/mouse, or sunitinib––orally at 35 mg/kg/mouse daily dose. Two groups received combination treatments with either bemcentinib + sunitinib or tilvestamab + sunitinib. IP injection of human IgG antibody served as a control for tilvestamab.

Tumor growth was evaluated by measuring the bioluminescence signal from 786‐0‐Luc cells using in vivo imaging system (IVIS) Lumina (PerkinElmer, Waltham, MA, USA) as described above after 3 and 5 weeks of the therapy. Results are presented as percentages of tumor growth, as measured by bioluminescence in relation to the size measured at the first time point.

### 2.9 Immunohistochemistry of Ki67, vimentin, and AXL

Animals were sacrificed by cervical dislocation on the day after last tumor imaging, and kidneys were dissected, washed in cold PBS (pH 7.5), fixed in 4% formaldehyde (Sigma) in PBS for 24 h at 4°C, dehydrated in a graded alcohol series and xylene, and finally embedded in paraffin wax.

FFPE sections from study II were stained for hematoxylin, Ki67, vimentin, and AXL by standardized immunohistochemistry (IHC) protocols. Briefly, 3 µm thick FFPE sections were deparaffinized with xylene and rehydrated in decreasing alcohol concentrations (100%—100%—96%—75%). Sections subjected to hematoxylin (Dako 3301) were incubated for 10–12 min before being rinsed with water. Sections for IHC were subjected to 25 min microwave treatment for antigen retrieval and submerged into pH 6 (S2369, Dako) or pH 9 citrate buffer solution (S2367, Dako), depending on which primary antibody was used for staining. To block endogenous peroxidase activity and non‐specific binding, peroxidase solution (S2023, Dako) and normal goat serum 10% in PBS were used for 8 and 30 min, respectively.

Sections were then incubated with primary antibodies for 1 h at RT. After a 5 min wash with PBST buffer, sections were incubated in labeled polymer HRP conjugated to goat anti‐mouse (Envision + system Dako, K4001) or HRP anti‐rabbit (k4003) for 30 min at RT. Few drops of 3,3’‐diaminobenzidine (DAB) (Dako, K3468) were then added to the sections, followed by counterstaining with hematoxylin (Dako). Finally, sections were dehydrated and cover‐slipped using non‐aqueous mounting medium. Stained slides were scanned in ScanScope (Aperio) and analyzed by ImageScope system (version 12.4.0.7018) with color deconvolution v9 algorithm (weak–medium–strong positive threshold: 100‐140‐180) for vimentin and quantifying nuclear v9 algorithm (weak–medium–strong positive threshold: 210‐188‐162) for Ki67.

### 2.10 Statistical analysis

Statistical analysis and graph construction were performed in SPSS Statistics Version 26, GraphPad Prism version 9.0.1, and Excel Microsoft Office 360. Results were presented as mean ± SD in in vitro and mean ± SE in in vivo experiments. One‐way ANOVA with Tukey's and Dunnett's post hoc test was used as statistical testing for in vitro and in vivo experiments, respectively. *p*‐value < 0.05 is considered statistically significant.

## 3 RESULTS

### 3.1 Prevention of AXL phosphorylation in 786‐0 cells through tilvestamab and bemcentinib

**FIGURE 1:**
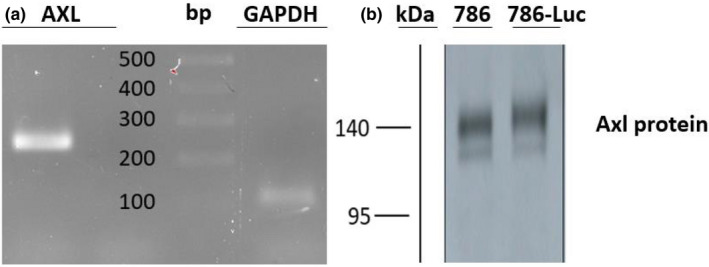
AXL expression in 786‐0 Luc cells at the mRNA and protein levels. (a) AXL expression in 786‐0‐Luc cells is detected at the mRNA level by RT‐qPCR. (b) AXL protein expression is confirmed by western blot analysis in both 786‐0 and 786‐0 Luc cells. DNA ladder; Thermo Scientific™ SM1173, Product Code. 11803983

First, we confirmed the AXL expression in 786‐0‐Luc cells at the mRNA and protein levels, as shown in Figure 1a and b.

**FIGURE 2:**
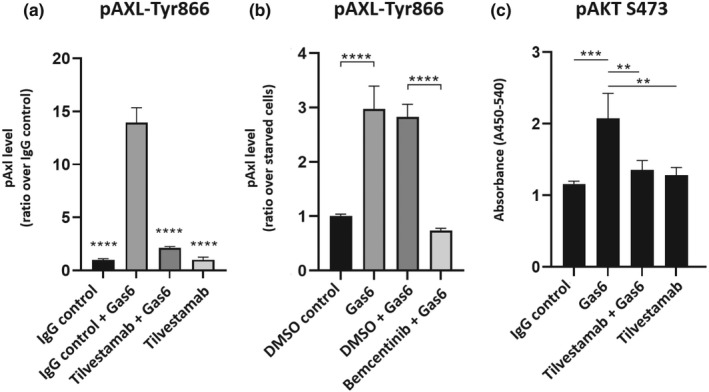
In vitro effects of specific inhibitors on AXL and AKT phosphorylation in 786‐0 cells. The pAXL levels, as measured in ELISA, are significantly reduced in tilvestamab (a) and bemcentinib (b)‐treated 786‐0 cells compared to their respective controls. Tilvestamab and bemcentinib results are reported as ratio over different controls. (c) Accordingly, pAKT levels are significantly reduced when cells are treated for 1 h with tilvestamab prior to Gas6 stimulation. ANOVA test with Tukey's post hoc is performed for statistical analysis. Bars presented with mean ± SD. ****p < 0.0001, ***p < 0.001, **p < 0.01

Thereafter, we proceed to evaluate the inhibitory effects of tilvestamab and bemcentinib on AXL phosphorylation in 786‐0 cells by ELISA, as depicted in Figure 2. Cell stimulation for 1 h in the presence of Gas6, a well characterized AXL ligand (Sasaki et al., 2006), resulted, as expected, in high AXL phosphorylation (pAXL). However, Gas6‐induced AXL phosphorylation was drastically inhibited by pretreatment of target cells with tilvestamab or bemcentinib, as compared to control IgG and DMSO, respectively (*p* < 0.0001), highlighted in Figure 2a and b. In particular, tilvestamab + Gas6 treatment resulted in approximately fivefold lower AXL activation as compared to control IgG + Gas6 (Figure 2a).

Phosphorylated AKT (pAKT), the downstream effector of AXL, was also evaluated. Accordingly, observed values were significantly lower in tilvestamab + Gas6 compared to 1 h Gas6‐treated cells (*p* < 0.01) (Figure 2c).

### 3.2 Inhibition of tumor growth by AXL inhibition in orthotopic RCC model

**FIGURE 3:**
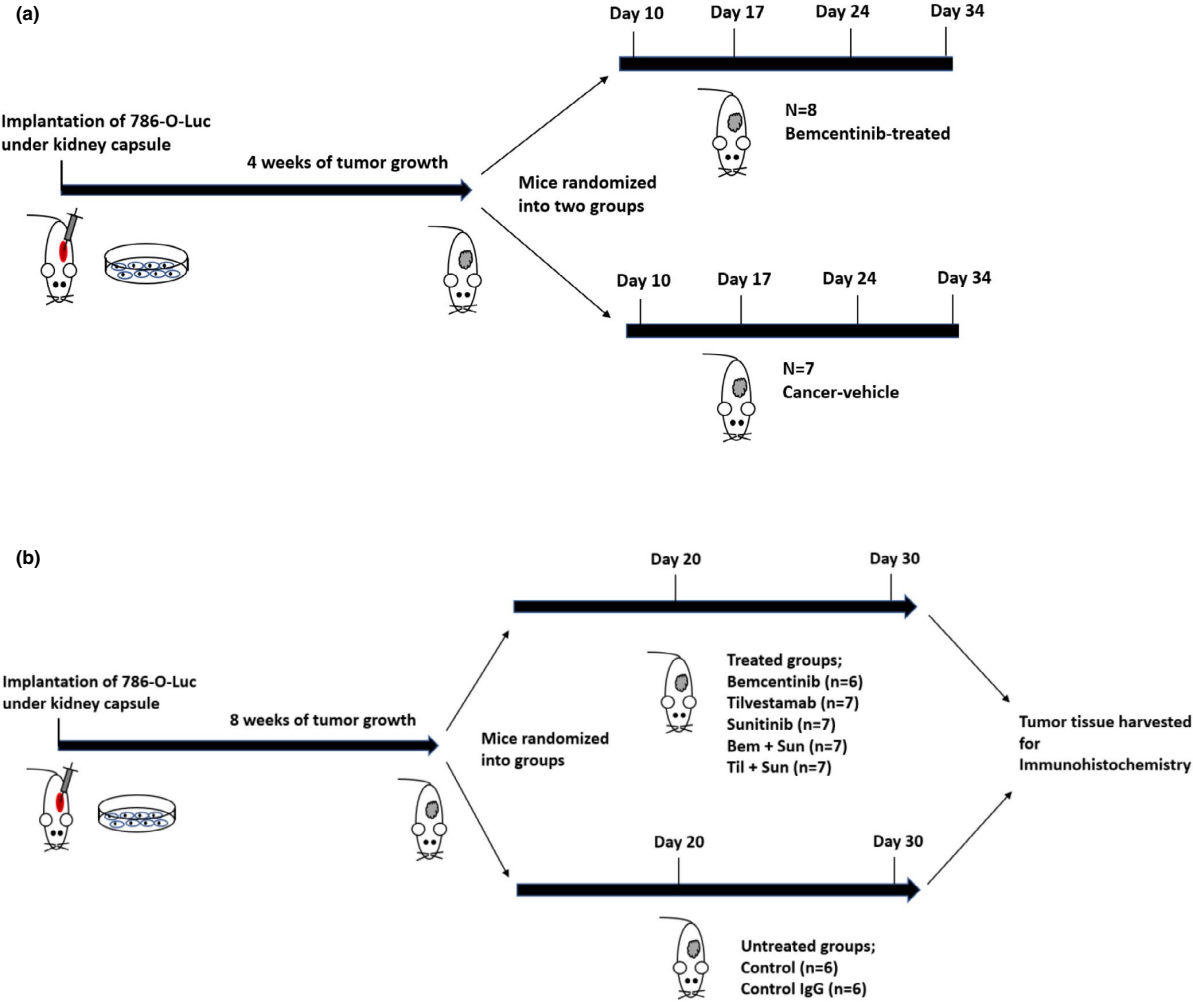
Schematic overview of the design of the two in vivo studies I (a) and II (b). 786‐0 Luc cells are implanted orthotopically in mice, and randomized into different treatment groups after initial tumor growth. (a) The effects of the treatment are measured by bioluminescence and 3D‐USG at days 10, 17, 24, and 34 after start of therapy in study. (b) Tumor growth in study II is measured by bioluminescence at days 20 and 30 after start of therapy. Tumor tissue is harvested at the time of sacrifice for immunohistochemistry analyses, as detailed in “Section 2”

The in vitro experiments described above, consistently confirmed the inhibitory potential of both tilvestamab and bemcentinib on AXL activation. Based on these results, we proceeded to in vivo studies in an orthotopic RCC murine model, as detailed in Figure 3.

**FIGURE 4:**
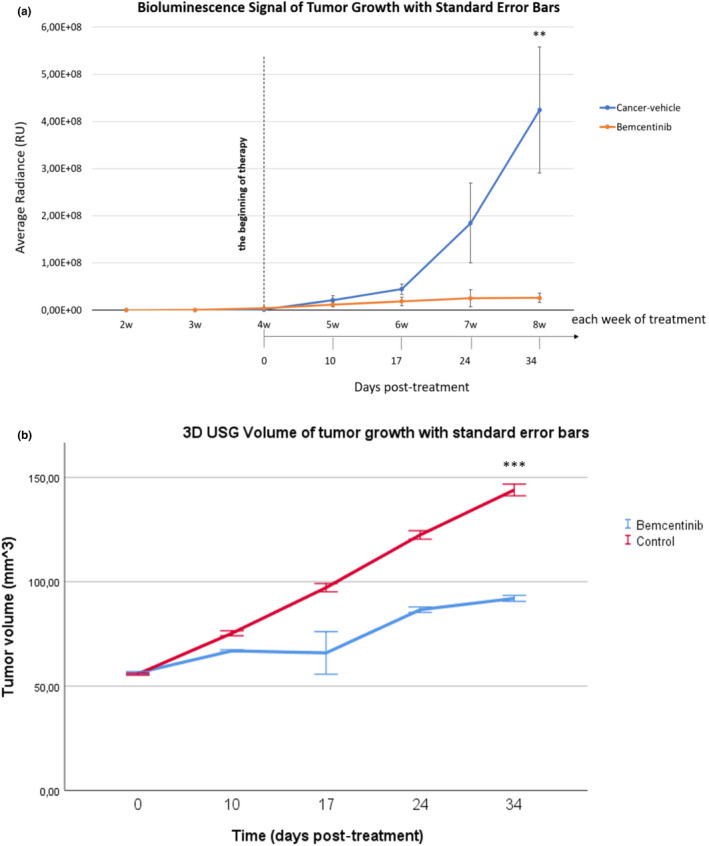
Bemcentinib inhibits progression of orthotopically implanted RCC 786‐0 tumors. The 786‐0‐Luc cells are orthotopically implanted in the kidneys, and treatment is initiated following detectable tumor growth. Antitumor effects of bemcentinib are measured and documented after 34 days administration by bioluminescence (a) and 3D USG (b), as detailed in “Section 2.” Graph made in IBM SPSS Statistics Version 26, and graph presented with mean ± SE. One‐way ANOVA test. **p < 0.05, ***p < 0.001

In the first study, tumor growth was monitored with bioluminescence signals using IVIS Lumina. After a 34‐day treatment we observed an average luminescence signal of 4.24 × 10^8^ RU ± 1.44 × 10^8^ (SE) in untreated animals, versus 2.58 × 10^7^ RU ± 1.05 × 10^7^ in bemcentinib‐treated mice, (*p* = 0.011) (Figure 4a). Accordingly, tumor volumes measured by ultrasound were also significantly higher in untreated (144 mm^3^ ± 2.79) compared to treated animals (92 mm^3^ ± 1.28) (*p* < 0.001) (Figure 4b).

**FIGURE 5:**
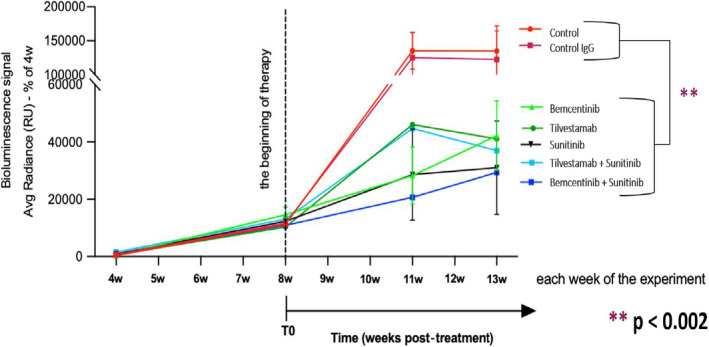
Comparative effects of AXL‐specific and multi‐targeted tyrosine kinase inhibitors, alone or in combination on the progression of orthotopically implanted 786‐0 cells. The 786‐0 cells are implanted orthotopically, and the indicated treatments are initiated following initial tumor growth. After a 34‐day administration, the effects of treatments in tumor growth are measured by bioluminescence, as detailed in “Section 2.” We obtained a clear tumor inhibitory effect of all treatment groups compared to the two controls. GraphPad Prism version 9.0.1 is used to generate the graph. ANOVA test with Dunnett's post hoc is applied. **p < 0.002

In the subsequent more extended second study, we tested the effects of bemcentinib and tilvestamab on orthotopic RCC growth in comparison with sunitinib, a well‐characterized multi‐target tyrosine kinase inhibitor, widely used in human RCC treatment (Ljungberg et al., 2020). As depicted in Figure 5, bioluminescence data show that bemcentinib, tilvestamab, and sunitinib significantly inhibited RCC growth down to about 1/3 of the volume, as compared to untreated or control IgG‐treated animals (*p* < 0.002).

Importantly, bemcentinib, tilvestamab, and sunitinib were similarly effective. Surprisingly, addition of sunitinib to bemcentinib or tilvestamab, did not significantly enhance their ability to inhibit tumor growth under our conditions. However, a tendency toward an additive effect of sunitinib to bemcentinib was observed after 5 weeks of co‐treatment (Figure 5). Longer follow‐up times could clarify this aspect.

Tumors of study II were further investigated using a histological approach. Hematoxylin staining of orthotopic 786‐0‐Luc RCC showed cancer cells, infiltrating lymphocytes, and areas of necrosis. IHC sections of these tumors revealed high expression of Ki67, a proliferation marker, and of vimentin, a classic RCC marker (Menon et al., 2019; Shi et al., 2015). Strong AXL expression was observed on the surface of cancer cells and in tumor stroma (data not shown). We could not quantify a visible effect of the various treatment modalities on these histological parameters probably due to the limited sensitivity of these analyses (data not shown).

## 4 DISCUSSION

RCC is a potentially fatal disease with worldwide increasing prevalence. Twenty percent to thirty percent of patients present mRCC at initial diagnosis, and a 5‐year relapse rate of 30%–40% has been observed in localized advanced RCC patients after surgical nephrectomy (Janowitz et al., 2013; Ljungberg et al., 2019). A third of mRCC patients do not respond to the standard treatment with tyrosine kinase inhibitor due to intrinsic resistance (Porta et al., 2012). Therapeutic options in advanced stage are limited. Therefore, novel therapeutic avenues are urgently needed.

Overexpression of AXL protein is highly correlated with advanced RCC stage and poor prognosis (Gustafsson et al., 2009). Mechanisms underlying this association are likely to be related to the ability of AXL to activate several different signal transduction pathways promoting tumor cell survival, proliferation, migration, and invasiveness as well as epithelial‐to‐mesenchymal (EMT) (Gay et al., 2017). In addition, a high AXL overexpression has been found during resistance to chemotherapy, including treatment with agents targeting epidermal growth factor receptor (EGFR), such as erlotinib, and with immune checkpoint inhibitors (Gay et al., 2017; Terry et al., 2021; Wu et al., 2014). These data suggest that AXL inhibitors might provide novel additional therapeutic options for the combined treatment of advanced RCC and various other cancers (Su et al., 2016; Zhang et al., 2012; Zhou et al., 2016).

Currently, cabozantinib, a multi‐target tyrosine kinase inhibitor, is the only drug able to block AXL used in mRCC treatment (Ljungberg et al., 2020). However, consistent with its multi‐target nature, cabozantinib administration has been associated with a variety of adverse side effects such as diarrhea, fatigue, nausea, vomiting, and weight loss that may limit its widespread use (Singh et al., 2017). Furthermore, as cabozantinib is not a specific inhibitor, it might not be as much effective as specific inhibitors in this context.

More recently, reagents with improved specificity have been developed. In particular, bemcentinib, a highly selective small molecule AXL inhibitor, was shown to block tumor growth in AXL‐expressing metastatic breast and esophageal cancer models (Holland et al., 2010; Yang et al., 2019). Moreover, the addition of bemcentinib has been reported to overcome resistance to EGFR inhibitors in experimental head and neck cancer cell line (Giles et al., 2013).

Based on these data, bemcentinib has entered multiple phase II clinical trials, either as single agent or in combination with targeted agents or chemotherapy in various types of cancers like triple‐negative breast cancer (TNBC), metastatic melanoma, and pancreatic cancer (Zhu et al., 2019). Most recently, a humanized anti‐AXL monoclonal antibody, tilvestamab, has successfully been developed by BerGenBio and is currently being tested for safety and dosage evaluation in healthy volunteers (Blø et al., 2020).

Indeed, AXL‐specific inhibition was previously shown to inhibit the growth of RCC xenografts but only based on subcutaneous injection of malignant cells (Yu et al., 2015). However, despite the striking association between AXL overexpression and poor clinical outcome in human RCC, AXL inhibitors have not been tested so far in orthotopic RCC models. Orthotopic models are considered superior to subcutaneous implanted models since they reflect more closely to biological tumor growth and metastasis in humans. Thus, orthotopic models could provide better prediction of drug therapy (Bibby, 2004).

In our study, we provide clear evidence of the ability of both bemcentinib and tilvestamab to significantly inhibit AXL activation and subsequent orthotopic tumor growth in vivo. Accordingly, and similar to clinical RCC specimens, the human RCC line 786‐0 used in the present study, displays a high AXL expression. Moreover, in our orthotopic model, tumors show high Ki67, AXL, and vimentin expression.

Our in vitro experiments show that both bemcentinib and tilvestamab effectively block AXL activation. Moreover, tilvestamab effectively block the downstream pAKT signaling in the 786‐0 cells used throughout the two in vivo studies. Most importantly, growth of orthotopic RCC is effectively inhibited by either reagent to about 1/3 of tumor size compared to controls. Notably, the addition of multi‐target TK inhibitor sunitinib to treatment might enhance the effects of these highly specific AXL inhibitors, especially in the case of bemcentinib, but our results do not allow any defined conclusions on this issue.

Considering the critical prognostic relevance of AXL expression in human RCC and the profound experimental in vivo effects, these data strongly support the potential clinical relevance of these compounds and pave the way toward their clinical testing in advanced RCC. Moreover, considering that activation of TAM kinases, including AXL, has been associated with suppression of antitumor immune responses (Holtzhausen et al., 2019), additional studies addressing the ability of the described reagents to promote the effectiveness of immunological checkpoint blockade are warranted.

## 5 CONCLUSION

Our data indicate that bemcentinib and tilvestamab as selective AXL inhibitors successfully inhibit the progression of an orthotopically implanted RCC and thus support the performance of clinical studies.

## CONFLICT OF INTEREST

Potential conflict of interest: M.B and G.G are employees from BerGenBio, Bergen, and have supplied the two AXL inhibiting agents.

## AUTHOR CONTRIBUTION

P.M, G.G, J.L, and H.P.M conceived and designed the research strategy. T.J.C, M.B.M, M.K, U.K, and M.B performed the experiments and analyzed the data. T.J.C, M.B.M, M.B, J.F, and T.O interpreted the results. T.J.C, M.B, and M.B.M prepared the figures. T.J.C and H.P.M drafted the manuscript. All authors edited and revised the manuscript. All authors approved the final version of the manuscript.
